# Data on DNA gel sample load, gel electrophoresis, PCR and cost analysis

**DOI:** 10.1016/j.dib.2017.11.082

**Published:** 2017-12-05

**Authors:** Ramona Kuhn, Jörg Böllmann, Kathrin Krahl, Isaac Mbir Bryant, Marion Martienssen

**Affiliations:** Brandenburg University of Technology Cottbus-Senftenberg, Institute of Environmental Technology, Chair of Biotechnology of Water Treatment, 03046 Cottbus, Germany

**Keywords:** Cost analysis, DNA sample load, Gel electrophoresis

## Abstract

The data presented in this article provide supporting information to the related research article “Comparison of ten different DNA extraction procedures with respect to their suitability for environmental samples” (Kuhn et al., 2017) [1]. In that article, we compared the suitability of ten selected DNA extraction methods based on DNA quality, purity, quantity and applicability to universal PCR. Here we provide the data on the specific DNA gel sample load, all unreported gel images of crude DNA and PCR results, and the complete cost analysis for all tested extraction procedures and in addition two commercial DNA extraction kits for soil and water.

**Specifications Table**

**Table:** 

Subject area	*Biology*
More specific subject area	*Molecular Biology*
Type of data	*Tables, figures, equations*
How data was acquired	*Bio View Biostep transilluminator*
Data format	*Raw and analyzed*
Experimental factors	*Sample were preserved at* −20 °C *before DNA extraction*
Experimental features	*DNA extraction, universal PCR, DNA visualization, cost analysis*
Data source location	*Cottbus, Germany*
Data accessibility	*Data is within this article*

**Value of the data**

• The data on the gel sample load are valuable to serve as indirect control for DNA quantification with fluorescence stain called PicoGreen.
• This data provide additional gel images of crude DNA and PCR of the tested DNA extraction procedures.
• The cost analysis of the DNA extraction procedures provided are valuable for further economical comparison.

## 1 Data

Table 1 presents the DNA sample load (in µL) necessary to visualize the crude DNA on the agarose gels. Different DNA loads were used in order to achieve comparable DNA concentrations ranging between 250 and 300 ng on the gel. Higher DNA loads were necessary for visualization on the agarose gels, especially for the crude DNA extracts from the Havel River sediment (procedure A, D, F, G, and H).

**Table 1:** Sample load in µL on the agarose gel for visualization of crude DNA extracts.

Extraction protocol according to first author		Origin of samples			
		Activated sludge	Havel River sediment	Anaerobic digestion sludge	Nitrifying sludge
A	Bourrain	4	15	5	8
B	Gabor harsh	2	8	5	8
C	Garbor soft	2	8	5	15
D	Shan	4	12	10	20
E	Orsini/Spica	4	8	6	15
F	Singka	4	12	15	15
G	Soya method	1	20	3	15
H	Tabatabaei	2	10	12	8
I	Tresse	1	6	6	10
J	Wilson	2	4	12	8

**Fig. 1:**
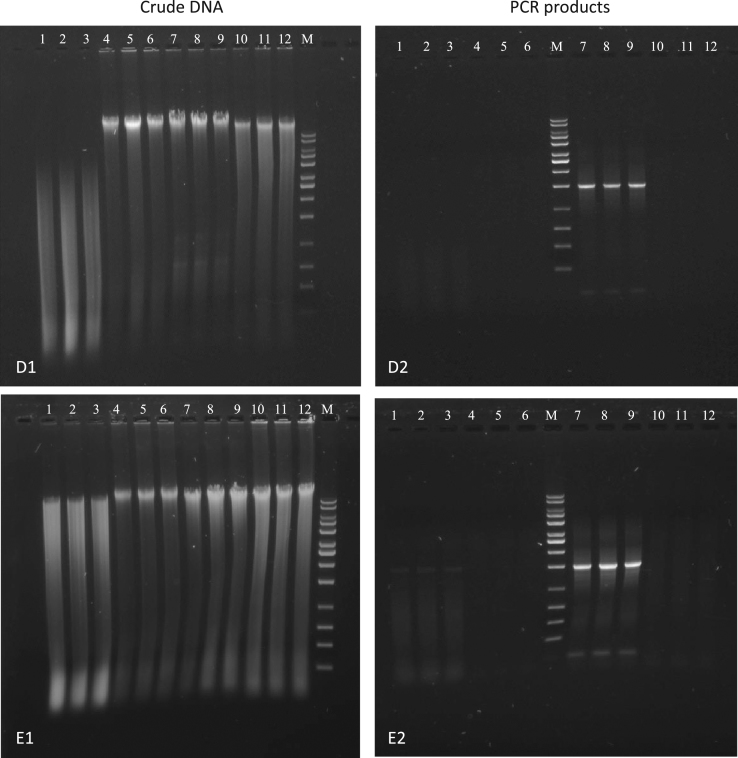
Agarose gel electrophoresis of crude DNA (D1 & E1) and universal PCR (D2 & E2) using universal primer set 27f and 1525r. D1 & D2: Procedure B (Gabor harsh). E1 & E2: Procedure C (Gabor soft). Lane declaration for all crude DNA and universal PCR gel images: lane 1 to 3 activated sludge; lane 4 to 6 Havel River sediment; lane 7 to 9 anaerobic digestion sludge; lane 10 to 12 nitrifying sludge; M in all gel images: 10 kb MassRuler DNA ladder.

**Fig. 2:**
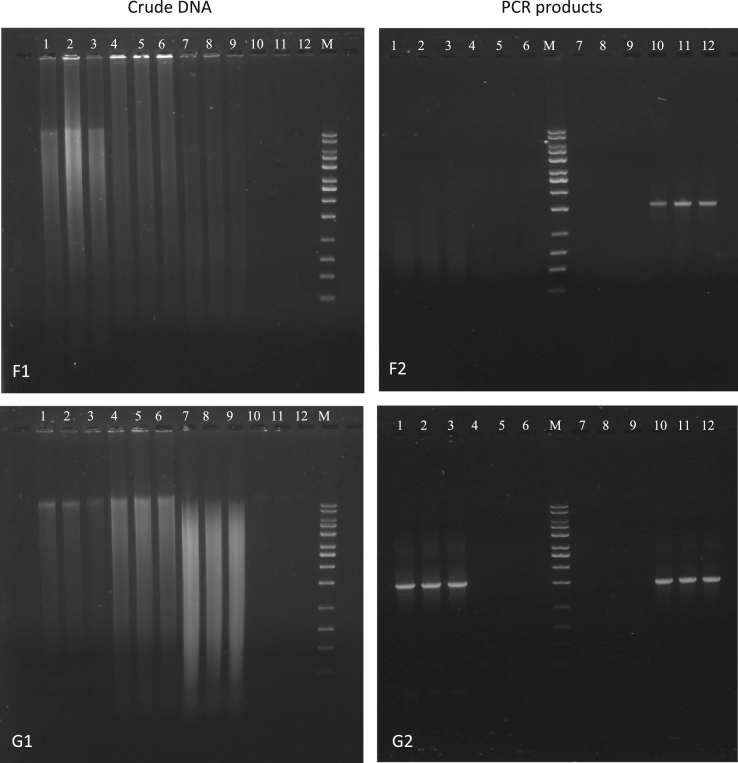
Agarose gel electrophoresis of crude DNA (F1 & G1) and universal PCR (F2 & G2) using universal primer set 27f and 1525r. F1 & F2: Procedure D (Shan). G1 & G2: Procedure E (Orsini & Romano-Spica). Lane declaration for all crude DNA and universal PCR gel images: lane 1 to 3 activated sludge; lane 4 to 6 Havel River sediment; lane 7 to 9 anaerobic digestion sludge; lane 10 to 12 nitrifying sludge; M in all gel images: 10 kb MassRuler DNA ladder.

**Fig. 3:**
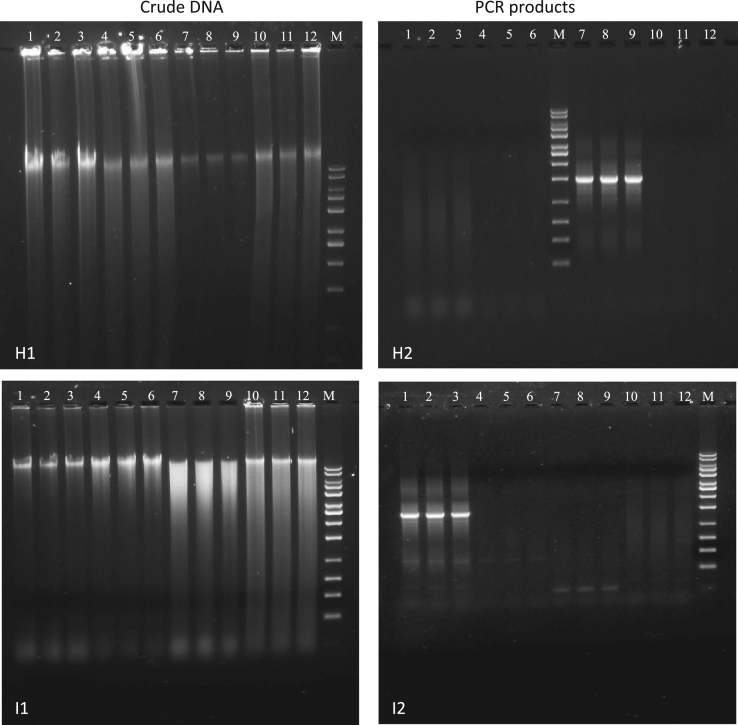
Agarose gel electrophoresis of crude DNA (H1, I1) and universal PCR (H2, I2) using universal primer set 27f and 1525r. H1 & H2: Procedure H (Tabatabaei). I1 & I2: Procedure I (Tresse). Lane declaration for all crude DNA and universal PCR gel images: lane 1 to 3 activated sludge; lane 4 to 6 Havel River sediment; lane 7 to 9 anaerobic digestion sludge; lane 10 to 12 nitrifying sludge; M in all gel images: 10 kb MassRuler DNA ladder.

**Fig. 4:**
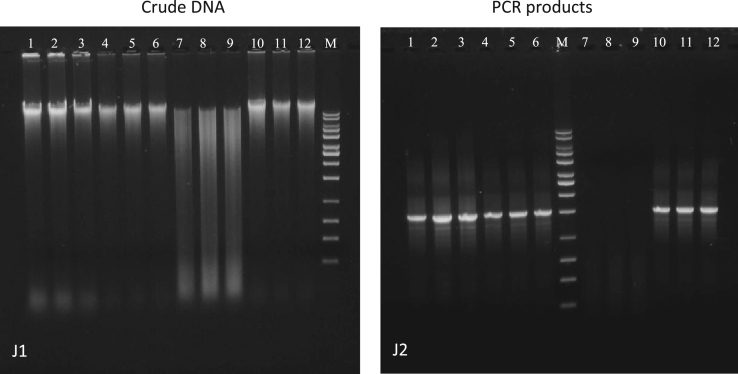
Agarose gel electrophoresis of crude DNA (J1) and universal PCR (J2) using universal primer set 27f and 1525r. G1 & G2: Procedure J (Wilson). Lane declaration for all crude DNA and universal PCR gel images: lane 1 to 3 activated sludge; lane 4 to 6 Havel River sediment; lane 7 to 9 anaerobic digestion sludge; lane 10 to 12 nitrifying sludge; M in all gel images: 10 kb MassRuler DNA ladder.

The visual DNA quality control of crude DNA extracts and PCR of procedures B, C, D, E, H, I and J is presented in Fig. 1, Fig. 2, Fig. 3, Fig. 4. The results for crude DNA extracts and PCR amplification of procedure B and C (method according to [2]) were almost similar. In both cases, intensive fragmentation was found for crude DNA extracts of the activated sludge and no distinct genomic DNA band was visible (Fig. 1, D1 & E1). The crude DNA of the sediment and anaerobic digestion sludge indicated a good quality with lower content of impurities, while the quality of the crude DNA for the nitrifying sludge was lower. A higher content of impurities was visible on both gel images. Positive PCR amplification was only feasible for the anaerobic digestion sludge and showed a very good quality of the amplicon (Fig. 1, D2 & E2).

The results for the crude DNA extracts of procedure D and E (method according to [3], [4]) were also almost similar (Fig. 2, F1 & G1). For procedure D, no distinct genomic DNA band was visible on the agarose gel but instead, fragmentation and higher content of undefined impurities (Fig. 2, F1). The pattern for the nitrifying sludge, especially, indicated complete failure of the extraction procedure. The gel image of the crude DNA extraction for procedure E occurred almost similar to procedure D with one exception. The crude DNA extract of the activated sludge showed a slight distinct genomic DNA band, however, the background staining indicated the presence of impurities (Fig. 2, G1). Nevertheless, positive PCR amplification was obtained for the crude DNA extract from activated sludge for procedure E (Fig. 2, G2). Surprisingly, positive amplification of the nitrifying sludge was also obtained for both procedure D and E (Fig. 2, F2).

The results of the crude DNA extracts of procedure H and I (method according to [5], [6]) are presented in Fig. 3. All crude DNA extracts of procedure H indicate a slight distinct genomic DNA band and higher content of impurities through background staining (Fig. 3, H1). Positive PCR amplification was only obtained for the crude DNA extract of the anaerobic digestion sludge. PCR amplification of the crude DNA extracts of the activated sludge, Havel River sediment and nitrifying sludge failed (Fig. 3, H2). The quality of crude DNA extracts of procedure I was different between the four environmental samples (Fig. 3, I1). A distinct genomic DNA band without higher content of visible impurities was obtained for the activated sludge. The degree of increased impurities occurred slightly for the crude DNA extracts of the Havel River sediment, but a distinct genomic DNA band was still good visible on the gel image. The crude DNA extract of the anaerobic digestion sludge showed higher content of DNA fragmentation as well as possible impurities in the background of the gel. Besides a distinct DNA band higher background smearing was also visible for the crude DNA extract of the nitrifying sludge. Positive PCR amplification was only obtained for the crude DNA extract of the activated sludge (Fig. 3, I2).

The results of the crude DNA extracts of procedure J are presented in Fig. 4 (method according to [7]). The gel image indicated distinct genomic DNA bands with lower content of background smearing for the activated sludge, Havel River sediment and the nitrifying sludge. A higher degree of possible DNA fragmentation and/or background impurities were observed for the crude DNA extract of the anaerobic digestion sludge (Fig. 4, J1). Positive PCR amplification was obtained from the activated sludge, Havel River sediment and the nitrifying sludge, while the amplification for the anaerobic digestion sludge failed (Fig. 4, J2).

The cost analysis of the ten DNA extraction procedures and the two commercial DNA extraction kits is presented in detail in Table 2, Table 3, Table 4, Table 5, Table 6, Table 7, Table 8, Table 9, Table 10, Table 11, Table 12, Table 13. Our cost analysis is based on cost estimation. Therefore a cost range between lowest and highest prices is presented. We assumed that the real extraction price will be in this cost range. The presented results show that every extraction procedure has its specific cost range, which is mainly dependent on the extraction time and therefore also on the cost of the laboratory staff. We calculated the lowest laboratory staff cost ranging between 3.65 € and 5.10 € for procedure J (Table 11), and the highest ranging between 8.68 and 12.15 for procedure A (Table 2). We calculated the lowest cost for the chemicals needed ranging between 0.13 € to 0.31 € for procedure D (Table 5) and the highest cost ranging between 0.47 € to 0.96 € for procedure I (Table 10). The cost for the other consumables such as gloves, tubes and tips were almost similar for all analyzed extraction procedures and extraction kits.

## 2 Experimental design, materials and methods

The sample preservation, DNA extraction, PCR performance and gel electrophoresis were described elsewhere [1]. For the cost analysis, a cost range was estimated ranging between minimum and maximum prices for all needed consumables. The number of required tubes and tips per extraction was counted. In all equations that follow, an index was included identifying low or high cost calculations, respectively. For clarification, the letter *x* represents all low cost calculations and the letter *y* represents all high cost calculations. The individual cost per chemical needed for every DNA extraction was calculated either with Eqs. (1) or (2), where *m*_*extraction*_ is the chemical weight required for a single DNA extraction and *m*_*total,fix cost*_ is the total weight corresponding to the fix cost. The individual cost for additional consumables such as gloves, tubes and/or tips was calculated either with Eqs. (3) or (4).

(1) $${\mathrm{Chemical}\;\mathrm{costs}}_{x}\left[€/\mathrm{prep}\right]=\frac{m_{\mathrm{extraction}}\times {\mathrm{Fix}\mathrm{cost}}_{x}}{m_{\mathrm{total},\;\mathrm{fix}\mathrm{cost}x}}$$

(2) $${\mathrm{Chemical}\;\mathrm{costs}}_{y}\left[€/\mathrm{prep}\right]=\frac{m_{\mathrm{extraction}}\times {\mathrm{Fix}\mathrm{cost}}_{y}}{m_{\mathrm{total},\;\mathrm{fix}\mathrm{cost}y}}$$

(3) $$\mathrm{Additional}\;\mathrm{concumable}\;{\mathrm{costs}}_{x}\left[€/\mathrm{prep}\right]=\frac{{\mathrm{Consumble}\mathrm{used}}_{\mathrm{extraction}}\times {\mathrm{Fix}\mathrm{cost}}_{x}}{{\mathrm{Total}\mathrm{consumable}}_{\mathrm{fix}\mathrm{cost}x}}$$

(4) $$\mathrm{Additional}\;\mathrm{concumable}\;{\mathrm{cost}s}_{y}\left[€/\mathrm{prep}\right]=\frac{{\mathrm{Consumble}\mathrm{used}}_{\mathrm{extraction}}\times {\mathrm{Fix}\mathrm{cost}}_{y}}{{\mathrm{Total}\mathrm{consumable}}_{\mathrm{fix}\mathrm{cost}y}}$$

The cost for the lab staff was calculated either with Eqs. (5) or (6). The calculation is based on a total of 12 extractions per staff and the individual extraction time of the tested extraction procedures.

(5) $${\mathrm{Lab}\mathrm{staff}}_{x}\left[€/\mathrm{prep}\right]=\left(\frac{{\mathrm{Cost}\mathrm{staff}}_{x}/\mathrm{Hour}}{12\;\mathrm{extractions}}\right)\times \left(\frac{\mathrm{Extraction}\mathrm{time}}{60\;\min}\right)$$

(6) $${\mathrm{Lab}\mathrm{staff}}_{y}\left[€/\mathrm{prep}\right]=\left(\frac{{\mathrm{Cost}\mathrm{staff}}_{x}/\mathrm{Hour}}{12\;\mathrm{extractions}}\right)\times \left(\frac{\mathrm{Extraction}\mathrm{time}}{60\;\min}\right)$$

The sum of total costs of chemicals was calculated either with Eqs. (7) or (8). The total costs of all additional consumables needed per extraction was calculated either with Eqs. (9) or (10). The final price per preparation was then calculated either with Eqs. (11) or (12) considering the cost for the lab staff, for all chemicals and additional consumables needed.

(7) $${\mathrm{Total}\;\mathrm{chemcial}\;\mathrm{costs}}_{x}\left[€/\mathrm{prep}\right]=\sum {\mathrm{Chemical}\;\mathrm{costs}}_{x}$$

(8) $${\mathrm{Total}\;\mathrm{chemcial}\;\mathrm{costs}}_{y}\left[€/\mathrm{prep}\right]=\sum {\mathrm{Chemical}\;\mathrm{costs}}_{y}$$

(9) $${\mathrm{Total}\;\mathrm{additional}\;\mathrm{consumables}\;\mathrm{costs}}_{x}\left[€/\mathrm{prep}\right]=\sum {\mathrm{Additional}\;\mathrm{consumable}\;\mathrm{costs}}_{x}$$

(10) $${\mathrm{Total}\;\mathrm{additional}\;\mathrm{consumables}\;\mathrm{costs}}_{y}\left[€/\mathrm{prep}\right]=\sum {\mathrm{Additional}\;\mathrm{consumable}\;\mathrm{costs}}_{y}$$

(11) $${\mathrm{Final}\;\mathrm{price}}_{x}\left[€/\mathrm{prep}\right]={\mathrm{Lab}\;\mathrm{staff}}_{x}+\sum {\mathrm{Chemical}\;\mathrm{costs}}_{x}+\sum {\mathrm{Additional}\;\mathrm{consumable}\;\mathrm{costs}}_{x}$$

(12) $${\mathrm{Final}\;\mathrm{price}}_{y}\left[€/\mathrm{prep}\right]={\mathrm{Lab}\;\mathrm{staff}}_{y}+\sum {\mathrm{Chemical}\;\mathrm{costs}}_{y}+\sum {\mathrm{Additional}\;\mathrm{consumable}\;\mathrm{costs}}_{y}$$

### 2.1 Cost analysis

See Table 2, Table 3, Table 4, Table 5, Table 6, Table 7, Table 8, Table 9, Table 10, Table 11, Table 12, Table 13.

**Table 2:** Cost analysis for DNA extraction procedure A (according to Bourrain et al., 1999).

**Consumables**	**Volumes**	**Units**	**Concentration**	**Volumes**	**/Weight**	**High costs**			**Low costs**			**Low cost**	**High cost**
						**Amount**	**Unit**	**Fix cost (€)**	**Amount**	**Unit**	**Fix cost (€)**	**per prep (€)**	**per prep (€)**
**Gloves (any size)**	1	pair	–	–	–	50	pair	8.20	50	pair	4.50	0.090	0.1640
**Tubes**	5	–	–	2.0	mL	500	pieces	11.9	1000	pieces	21.90	0.1095	0.1190
**Tips**	12	–	–	1000	µL	500	pieces	5.08	1000	pieces	7.70	0.0924	0.1218
**Tips**	1	–	–	200	µL	500	pieces	5.40	1000	pieces	8.19	0.0082	0.0108
**Lysozyme buffer**	0.75	mL	0.15 M NaCl	6,6	mg	500	g	15.84	1000	g	24.19	0.0002	0.0002
			0.1 M Na2EDTA	27.9	mg	100	g	23.50	1000	g	59.70	0.0017	0.0066
			15 mg mL-1 Lysozyme	15.0	mg	1.0	g	23.89	10	g	96.04	0.1441	0.3584
**SDS solution**	0.75	mL	0.1 M NaCl	4.4	mg	500	g	15.84	1000	g	24.19	0.0001	0.0001
			0.5 M Tris–HCl	45.4	mg	500	g	93.40	1000	g	128.00	0.0058	0.0085
			w/v 10% SDS	0.075	mg	100	g	16.56	1000	g	56.48	0.0000	0.0000
**Tris–HCl saturated phenol**	1.0	mL	0.1 M Tris–HCl	12.1	mg	500	g	93.40	1000	g	128.00	0.0016	0.0023
			Phenol	1.0	g	100	g	18.00	1000	g	64.40	0.0644	0.1800
**Phenol:Chloroform:Isoamyl**	1.0	mL	25′ Phenol	0.5	g	100	g	18.00	1000	g	64.40	0.0322	0.0900
**(25:24:1 v/v)**			24′ Chloroform	0.48	mL	500	mL	50.62	2500	mL	100.66	0.0193	0.0486
			1′ Isoamyl	0.02	mL	25	mL	13.92	1000	mL	108.00	0.0022	0.0111
**Chloroform:Isoamyl**	1.0	mL	24′ Chloroform	0.96	mL	500	mL	50.62	2500	mL	100.66	0.0387	0.0972
**(24:1 v/v)**			1′ Isoamyl	0.04	mL	25	mL	13.92	1000	mL	108.00	0.0043	2.2E-05
**Isopropanol**	1.0	mL	100%	2.0	mL	1000	mL	30.30	2500	mL	61.70	0.0494	6.1E-02
**TE buffer**	0.1	mL	10 mM Tris–HCl	0.12	mg	500	g	93.40	1000	g	128.00	1.6E-05	2.3E-05
			1 mM EDTA	0.03	mg	100	g	34.08	1000	g	245.23	7.2E-06	1.0E-05
**RNaseA treatment**	5.0	µL	0.2 µg µL-1	1.0	µg	250	mg	94.40	1000	mg	292.00	0.0003	3.8E-04
**Extracted samples**	12					–		–	–		–	–	–
**Extraction time**	250	min				–		–	–		–	–	–
**Lab staff (per hour)**						–		35.00	–		25.00	–	–
**Lab staff (€/extraction)**												**8.68**	**12.15**
**Chemicals (€/extraction)**												**0.36**	**0.86**
**Gloves, tubes, tips (€/extraction)**												**0.30**	**0.42**
**Final price per extraction including extraction time, lab staff and all consumables (€)**												**9.34**	**13.43**

**Table 3:** Cost analysis for DNA extraction procedure B (according to Gabor et al. [2]; harsh method).

**Consumables**	**Volumes**	**Units**	**Concentration**	**Volumes**	**/Weight**	**High costs**			**Low costs**			**Low cost**	**High cost**
						**Amount**	**Unit**	**Fix cost (€)**	**Amount**	**Unit**	**Fix cost (€)**	**per Prep (€)**	**per Prep (€)**
**Gloves (any size)**	1	pair	–	–	–	50	pair	8.20	50	pair	4.50	0.0900	0.1640
**Tubes**	3	–	–	2.0	mL	500	pieces	11.90	1000	pieces	21.90	0.0657	0.0714
**Tips**	10			1000	µL	500	pieces	5.08	1000	pieces	7.70	0.0770	0.1015
**Tips**	4			200	µL	500	pieces	5.40	1000	pieces	8.19	0.0328	0.0432
**Tips**	1			10	µL	1000	pieces	27.14	2000	pieces	43.42	0.0217	0.0271
**Silica beads**	0.1	mm	–	700	mg	1000	g	24.30	25000	g	202.00	0.0057	0.0170
**Lysozyme buffer**	1.25	mL	100 mM Tris	15.1	mg	500	g	93.40	1000	g	128.00	0.0019	0.0028
		mL	100 mM sodium EDTA	46.5	mg	100	g	23.50	1000	g	59.70	0.0028	0.0109
			100 M NaCl	109.6	mg	500	g	15.84	1000	g	24.19	0.0027	0.0035
			1% w/v CTAB	12.5	µg	100	g	22.64	1000	g	89.11	0.0011	0.0028
**Lysozyme**	0.04	mL	50 mg mL^−1^	2.0	mg	1.0	g	23.89	10	g	96.04	0.0192	0.0478
**Proteinase K**	0.01	mL	10 mg mL^−1^	0.1	mg	0.1	g	67.68	0.5	g	259.62	0.0519	0.0677
**SDS**	0.2	mL	w/v 20%	0.04	mg	100	g	16.56	1000	g	56.48	2.3E-06	6.6E-06
**Chloroform (1:1 v/v)**	1.0	mL	100%	1.0	mL	500	mL	50.62	2500	mL	100.66	0.0403	0.1012
**Isopropanol (0.6:1 v/v)**	0.6	mL	100%	0.6	mL	1000	mL	30.30	2500	mL	61.70	0.0148	0.0182
**Ethanol**	0.5	mL	70%	0.375	mL	250	mL	47.56	2500	mL	246.58	0.0345	0.0666
**TE buffer**	0.1	mL	10 mM Tris–HCl	0.12	mg	500	g	93.40	1000	g	128.00	1.6E-05	2.3E-05
			1 mM EDTA	0.03	mg	100	g	34.08	1000	g	245.23	7.2E-06	1.0E-05
**Extracted samples**	12					–		–	–		–	–	–
**Extraction time**	235	min				–		–	–		–	–	–
**Lab staff (per hour)**						–		35.00	–		25.00	–	–
**Lab staff (€/extraction)**												**8.16**	**11.42**
**Chemicals (€/extraction)**												**0.17**	**0.34**
**Gloves, tubes, tips (€/extraction)**												**0.29**	**0.41**
**Final price per extraction including extraction time, lab staff and all consumables (€)**												**8.62**	**12.17**

**Table 4:** Cost analysis for DNA extraction procedure C (according to Gabor et al. [2]; soft method).

**Consumables**	**Volumes**	**Units**	**Concentration**	**Volumes**	**/Weight**	**High costs**			**Low costs**			**Low cost**	**High Cost**
						**Amount**	**Unit**	**Fix cost (€)**	**Amount**	**Unit**	**Fix cost (€)**	**per Prep (€)**	**per Prep (€)**
**Gloves (any size)**	1	pair	–	–	–	50	pair	8.20	50	pair	4.50	0.090	0.164
**Tubes**	3	–	–	2.0	mL	500	pieces	11.90	1000	pieces	21.90	0.066	0.071
**Tips**	10			1000	µL	500	pieces	5.08	1000	pieces	7.70	0.077	0.102
**Tips**	4			200	µL	500	pieces	5.40	1000	pieces	8.19	0.033	0.043
**Tips**	1			10	µL	1000	pieces	27.14	2000	pieces	43.42	0.022	0.027
**Silica beads**	0.1	mm	ID	700	mg	1000	g	24.30	25000	g	202.00	0.0057	0.0170
**Lysozyme buffer**	1.25	mL	100 mM Tris	15.1	mg	500	g	93.40	1000	g	128.00	0.0019	0.0028
		mL	100 mM sodium EDTA	46.5	mg	100	g	23.50	1000	g	59.70	0.0028	0.0109
			100 M NaCl	109.6	mg	500	g	15.84	1000	g	24.19	0.0027	0.0035
			1% w/v CTAB	12.5	µg	100	g	22.64	1000	g	89.11	0.0011	0.0028
**Lysozyme**	0.04	mL	50 mg mL-1	2.0	mg	1.0	g	23.89	10	g	96.04	0.0192	0.0478
**Proteinase K**	0.01	mL	10 mg mL-1	0.1	mg	0.1	g	67.68	0.5	g	259.62	0.0519	0.0677
**SDS**	0.2	mL	w/v 20%	0.04	mg	100	g	16.56	1000	g	56.48	2.3E-06	6.6E-06
**Chloroform (1:1 v/v)**	1.0	mL	100%	1.0	mL	500	mL	50.62	2500	mL	100.66	0.0403	0.1012
**Isopropanol (0.6:1 v/v)**	0.6	mL	100%	0.6	mL	1000	mL	30.30	2500	mL	61.70	0.0148	0.0182
**Ethanol**	0.5	mL	70%	0.375	mL	250	mL	47.56	2500	mL	246.58	0.0345	0.0666
**TE buffer**	0.1	mL	10 mM Tris–HCl	0.12	mg	500	g	93.40	1000	g	128.00	1.6E-05	2.3E-05
			1 mM EDTA	0.03	mg	100	g	34.08	1000	g	245.23	7.2E-06	1.0E-05
**Extracted samples**	12					–		–	–		–	–	–
**Extraction time**	230	min				–		–	–		–	–	–
**Lab staff (per hour)**						–		35.00	–		25.00	–	–
**Lab staff (€/extraction)**												**7.99**	**11.18**
**Chemicals (€/extraction)**												**0.17**	**0.34**
**Gloves, tubes, tips (€/extraction)**												**0.29**	**0.41**
**Final price per extraction including extraction time, lab staff and all consumables (€)**												**8.45**	**11.93**

**Table 5:** Cost analysis for DNA extraction procedure D (according to Shan et al. [3]).

**Consumables**	**Volumes**	**Units**	**Concentration**	**Volumes**	**/Weight**	**High costs**			**Low costs**			**Low cost**	**High cost**
						**Amount**	**Unit**	**Fix cost (€)**	**Amount**	**Unit**	**Fix cost (€)**	**per Prep (€)**	**per Prep (€)**
**Gloves (any size)**	1	pair	–	–	–	50	pair	8.20	50	pair	4.50	0.0900	0.1640
**Tubes**	3	–	–	2.0	mL	500	pieces	11.90	1000	pieces	21.90	0.0657	0.0714
**Tips**	8			1000	µL	500	pieces	5.08	1000	pieces	7.70	0.0616	0.0812
**Tips**	2			200	µL	500	pieces	5.40	1000	pieces	8.19	0.0164	0.0216
**Tips**	1			10	µL	1000	pieces	27.14	2000	pieces	43.42	0.0217	0.0271
**TENP Puffer**	0.4	mL	50 mM Tris	2.42	mg	500	g	93.40	1000	g	128.00	0.0003	0.0005
			20 mM EDTA	2.34	mg	100	g	34.08	1000	g	245.23	0.0006	0.0008
			100 mM NaCl	2.34	mg	500	g	15.84	1000	g	24.19	0.0001	0.0001
			10 mg mL-1 PVP	4.00	mg	100	g	45.30	1000	g	224.00	0.0009	0.0018
**SDS**	50	µL	w/v 20%	10.0	µg	10]0	g	16.56	1000	g	56.48	5.6E-07	1.7E-06
**CTAB Puffer**	0.5	mL	0,7 M NaCl	20.5	mg	500	g	15.84	1000	g	24.19	0.0005	0.0006
			10% CTAB	50.0	µg	100	g	22.64	1000	g	89.11	4.5E-06	1.1E-05
**KH**_**2**_**PO**_**4**_	0.25	mL	240 mM	8.16	mg	250	g	19.66	1000	g	56.66	4.6E-07	0.0006
**Phenol:Chloroform:Isoamyl**	1.0	mL	100 mM Tris	12.1	mg	500	g	93.40	1000	g	128.00	0.0016	0.0023
**(25:24:1 v/v)**			Phenol	0.50	g	100	g	18.00	1000	g	64.40	0.0322	0.0900
			Chloroform	0.48	mL	500	mL	50.62	2500	mL	100.66	0.0193	0.0486
			Isoamyl	0.02	mL	25	mL	13.92	1000	mL	108.00	0.0022	0.0111
**Chloroform:Isoamyl**	1.0	mL	Chloroform	0.96	mL	500	mL	50.62	2500	mL	100.66	0.0387	0.0972
**(24:1 v/v)**			Isoamyl	0.04	mL	25	mL	13.92	1000	mL	108.00	0.0043	0.0223
**Isopropanol**	1.0	mL	100%	1.0	mL	1000	mL	30.30	2500	mL	61.70	0.0247	0.0303
**TE buffer**	0.1	mL	10 mM Tris–HCl	0.12	mg	500	g	93.40	1000	g	128.00	1,6E-05	2.3E-05
			1.0 mM EDTA	0.03	mg	100	g	34.08	1000	g	245.23	7.2E-06	1.0E-05
**Extracted samples**	12					–		–	–			–	–
**Extraction time**	210	min				–		–	–		–	–	–
**Lab staff (per hour)**						–		35.00	–		25.00	–	–
**Lab staff (€/extraction)**												**7.29**	**10.21**
**Chemicals (€/extraction)**												**0.13**	**0.31**
**Gloves, tubes, tips (€/extraction)**												**0.26**	**0.37**
**Final price per extraction including extraction time, lab staff and all consumables (€)**												**7.67**	**10.88**

**Table 6:** Cost analysis for DNA extraction procedure E (according to Orsini and Romano-Spica [4]).

**Consumables**	**Volumes**	**Units**	**Concentration**	**Volumes**	**/Weight**	**High costs**			**Low costs**			**Low cost**	**High cost**
						**Amount**	**Unit**	**Fix cost (€)**	**Amount**	**Unit**	**Fix cost (€)**	**per Prep (€)**	**per Prep (€)**
**Gloves (any size)**	1	pair	–	–	–	50	pair	8.20	50	pair	4.50	0.0900	0.1640
**Tubes**	2	–	–	2.0	mL	500	pieces	11.90	1000	pieces	21.90	0.0438	0.0476
**Tips**	9		–	1000	µL	500	pieces	5.08	1000	pieces	7.70	0.0693	0.0914
**Tips**	3		–	200	µL	500	pieces	5.40	1000	pieces	8.19	0.0246	0.0324
**Tips**	0		–	10	µL	1000	pieces	27.14	2000	pieces	43.42	0,0000	0.0000
**Wash solution**	1.0	mL	50 mM Tris–HCl	6.1	mg	500	g	93.40	1000	g	128.00	0.0008	0.0011
			25 mM EDTA	7.3	mg	100	g	34.08	1000	g	245.23	0.0018	0.0025
			0.1% w/v SDS	1.0	µg	100	g	16.56	1000	g	56.48	5.6E-08	1.7E-07
			0.1% w/v PVP	1.0	µg	100	g	45.30	1000	g	224.00	2.2E-07	4.5E-07
**Lysis buffer**	0.1	mL	50 mM Tris–HCl	0.61	mg	500	g	93.40	1000	g	128.00	7.8E-05	1.1E-04
			25 mM EDTA	0.73	mg	100	g	34.08	1000	g	245.23	1.8E-04	2.5E-04
			3% w/v SDS	30.0	µg	100	g	16.56	1000	g	56.48	1.7E-06	5.0E-06
			1.2% w/v PVP	12.0	µg	100	g	45.30	1000	g	224.00	2.7E-06	5.4E-06
**Extraction buffer**	0.8	mL	10 mM Tris–HCl	9.7	mg	500	g	93.40	1000	g	128.00	0.0012	0.0018
			1 mM EDTA	0.23	mg	100	g	34.08	1000	g	245.23	0.0001	0.0001
			0.3 M NaOAc	19.7	mg	250	g	22.47	1000	g	56.30	0.0011	0.0018
			1.2% PVP	9.6	µg	100	g	45.30	1000	g	224.00	2.2E-06	4.3E-06
**Phenol:Chloroform**	1.0	mL	Phenol	0.5	g	100	g	18.00	1000	g	64.40	0.0322	0.0900
**(1:1 v/v)**			Chloroform	0.5	mL	500	mL	50.62	2500	mL	100.66	0.0201	0.0506
**Sodiumacetate**	0.08	mL	3 M	19.7	mg	250	g	22.47	1000	g	56.30	0.0011	0.0018
**Isopropanol**	0.9	mL	100%	0.9	mL	1000	mL	30.30	2500	mL	61.70	0.0222	0.0273
**Ethanol**	2.0	mL	70%	1.4	mL	250	mL	47.56	2500	mL	246.58	0.1381	0.2663
**TE buffer**	0.1	mL	10 mM Tris–HCl	0.12	mg	500	g	93.40	1000	g	128.00	1.6E-05	2.3E-05
			1.0 mM EDTA	0.03	mg	100	g	34.08	1000	g	245.23	7.2E-06	1.0E-05
**Extracted samples**	12					–		–	–		–	–	–
**Extraction time**	150	min				–		–	–		–	–	–
**Lab staff (per hour)**						–		35.00	–		25.00	–	–
**Lab staff (€/extraction)**												**5.21**	**7.29**
**Chemicals (€/extraction)**												**0.22**	**0.44**
**Gloves, tubes, tips (€/extraction)**												**0.23**	**0.34**
**Final price per extraction including extraction time, lab staff and all consumables (€)**												**5.65**	**8.07**

**Table 7:** Cost analysis for DNA extraction procedure F (according to Singka et al., 2012).

**Consumables**	**Volumes**	**Units**	**Concentration**	**Volumes**	**/Weight**	**High costs**			**Low costs**			**Low cost**	**High cost**
						**Amount**	**Unit**	**Fix cost (€)**	**Amount**	**Unit**	**Fix cost (€)**	**per Prep (€)**	**per Prep (€)**
**Gloves (any size)**	1	pair	–	–	–	50	pair	8.20	50	pair	4.50	0,.900	0.1640
**Tubes**	4	–	–	1.5	mL	500	pieces	8.20	1000	pieces	14.90	0.0596	0.0656
**Tips**	12		–	1000	µl	500	pieces	5.08	1000	pieces	7.70	0.0924	0.1218
**Tips**	2		–	200	µL	500	pieces	5.40	1000	pieces	8.19	0.0164	0.0216
**Glass beads 0.1 mm**	0.5	g	–	5.0	g	1000	g	24.30	25000	g	202.00	0.0404	0.1215
**CTAB extraction buffer**	0.5	mL	0.7 M NaCl	10.2	mg	500	g	15.84	1000	g	24.19	0.0002	0.0003
**(1:1 v/v) 10% w/v (CTAB in NaCl)**			10% w/v CTAB	2,5	µg	100	g	22.64	1000	g	89.11	2.2E-07	5.7E-07
**to KH**_**2**_**PO**_**4**_			240 mM KH2PO4	8.2	mg	250	g	19.66	1000	g	56.66	0.0005	0.0006
**Phenol:Chloroform:Isoamyl**	1.0	mL	25′ Phenol	0.5	g	100	g	18.00	1000	g	64.40	0.0322	0.0900
**(25:24:1 v/v)**			24′ Chloroform	0.48	mL	500	mL	50.62	2500	mL	100.66	0.0193	0.0486
			1′ Isoamyl	0.02	mL	25	mL	13.92	1000	mL	108.00	0.0022	0.0111
**Chloroform:Isoamyl**	0.5	mL	24' Chloroform	0.48	mL	500	mL	50.62	2500	mL	100.66	0.0193	0.0486
**(24:1 v/v)**			1′ Isoamyl	0.02	mL	25	mL	13.92	1000	mL	108.00	0.0022	0.0111
**Sodium acetate (0.1:1 v/v)**	0.05	mL	3 M	12.3	mg	250	g	22.47	1000	g	56.30	0.0007	0.0011
**Isopropanol (0.6: 1 v/v)**	0.3	mL	100%	0.3	mL	1000	mL	30.30	2500	mL	61.70	0.0074	0.0091
**Ethanol**	1.5	mL	70%	1.05	mL	250	mL	47.56	2500	mL	246.58	0.1036	0.1998
**TE buffer**	0.1	mL	10 mM Tris–HCl	0.12	mg	500	g	93.40	1000	g	128.00	1.6E-05	2.3E-05
			1 mM EDTA	0.03	mg	100	g	34.08	1000	g	245.23	7.2E-06	1.0E-05
**Extracted samples**	12					–			–			–	–
**Extraction time**	195	min				–		–	–		–	–	–
**Lab staff (per hour)**						–		35.00	–		25.00	–	–
**Lab staff (€/extraction)**												**6.77**	**9.48**
**Chemicals (€/extraction)**												**0.19**	**0.42**
**Gloves, tubes, tips (€/extraction)**												**0.30**	**0.49**
**Final price per extraction including extraction time, lab staff and all consumables (€)**												**7.26**	**10.39**

**Table 8:** Cost analysis for DNA extraction procedure G (according to Saxony State Method).

**Consumables**	**Volumes**	**Units**	**Concentration**	**Volumes**	**/Weight**	**High costs**			**Low costs**			**Low cost**	**High Cost**
						**Amount**	**Unit**	**Fix cost (€)**	**Amount**	**Unit**	**Fix cost (€)**	**per Prep (€)**	**per Prep (€)**
**Gloves (any size)**	1	pair	–	–	–	50	pair	8.20	50	pair	4.50	0.0900	0.1640
**Tubes**	3	–	–	2.0	mL	500	pieces	11.90	1000	pieces	21.90	0.0657	0.0714
	1	–	–	1.5	mL	500	pieces	8.20	1000	pieces	14.90	0.0149	0.0164
**Tips**	13		–	1000	µL	500	pieces	5.08	1000	pieces	7.70	0.1001	0.1320
**Tips**	1		–	200	µL	500	pieces	5.40	1000	pieces	8.19	0.0082	0.0108
**Tips**	1		–	10	µL	1000	pieces	27.14	2000	pieces	43.42	0.0217	0.0271
**Extraction buffer**	1.0	mL	2% w/v CTAB	20.0	µg	100	g	22.64	1000	g	89.11	1.8E-06	4.5E-06
			0.1 M Tris–HCl	12.1	mg	500	g	93.40	1000	g	128.00	0.0016	0.0023
			0.02 M EDTA	5.8	mg	100	g	34.08	1000	g	245.23	0.0014	0.0020
			1.4 M NaCl	81.8	mg	500	g	15.84	1000	g	24.19	0.0020	0.0026
**RNase A**	0.02	mL	20 mg mL-1	0.4	mg	250	mg	94.40	1000	mg	292.00	0.1168	0.1510
**Chloroform**	0.75	mL	100%	0.75	mL	500	mL	50.62	2500	mL	100.66	0.0302	0.0759
**Precipitation solution**	1.0	mL	0.5% w/v CTAB	0.5	µg	100	g	22.64	1000	g	89.11	4.5E-08	1.1E-07
			40 mM NaCL	2.3	mg	500	g	15.84	1000	g	24.19	0.0001	0.0001
**NaCl**	0.35	mL	1.2 M NaCl	24.5	mg	500	g	15.84	1000	g	24.19	0.0006	0.0008
**Chloroform**	0.35	mL	100%	0.35	mL	500	mL	50.62	2500	mL	100.66	0.0141	0.0354
**Isopropanol (0.6:1 v/v)**	0.15	mL	100%	0.15	mL	250	mL	47.56	2500	mL	246.58	0.0148	0.0285
**Ethanol**	1.5	mL	70%	1.05	mL	250	mL	47.56	2500	mL	246.58	0.1036	0.1998
**TE buffer**	0.1	mL	10 mM Tris–HCl	0.12	mg	500	g	93.40	1000	g	128.00	1.6E-05	2.3E-05
			1.0 mM EDTA	0.03	mg	100	g	34.08	1000	g	245.23	7.2E-06	1.0E-05
**Extracted samples**	12					–		–	–		–	–	–
**Extraction time**	175	min				–		–	–		–	–	–
**Lab staff (per hour)**						–		35.00	–		25.00	–	–
**Lab staff (€/extraction)**												**6.08**	**8.51**
**Chemicals (€/extraction)**												**0.29**	**0.50**
**Gloves, tubes, tips (€/extraction)**												**0.30**	**0.42**
**Final price per extraction including extraction time, lab staff and all consumables (€)**												**6.66**	**9.43**

**Table 9:** Cost analysis for DNA extraction procedure H (according to Tabatabaei et al. [5]).

**Consumables**	**Volumes**	**Units**	**Concentration**	**Volumes**	**/Weight**	**High costs**			**Low costs**			**Low cost**	**High cost**
						**Amount**	**Unit**	**Fix cost (€)**	**Amount**	**Unit**	**Fix cost (€)**	**per Prep (€)**	**per Prep (€)**
**Gloves (any size)**	1	pair	–	–	–	50	pair	8.20	50	pair	4.50	0.0900	0.1640
**Tubes**	3	–	–	2.0	mL	500	pieces	11.90	1000	pieces	21.90	0.0657	0.0714
**Tips**	12		–	1000	µL	500	pieces	5.08	1000	pieces	7.70	0.0924	0.1218
**Tips**	1		–	200	µL	500	pieces	5.40	1000	pieces	8.19	0.0082	0.0108
**Tips**	0		–	10	µL	1000	pieces	27.14	2000	pieces	43.42	0.0000	0.0000
**EDTA**	0.4	mL	0.5 EDTA	58.4	mg	100	g	34.08	1000	g	245.23	0.0143	0.0199
**Lysis buffer**	0.4	mL	10 mM Tris	0.48	mg	500	g	93.40	1000	g	128.00	0.0001	0.0001
			1 mM EDTA	0.12	mg	100	g	34.08	1000	g	245.23	3.E-05	4.E-05
			2 mg mL-1 Lysozyme	0.80	mg	1,0	g	23.89	10	g	96.04	0.0077	0.0191
**SDS**	0.05	mL	10% w/v	0.005	mg	100	g	16.56	1000	g	56.48	2.8E-07	8.3E-07
**Phenol:Chloroform**	0.8	mL	Phenol	0.4	g	100	g	18.00	1000	g	64.40	0.0258	0.0720
**(1:1 v/v)**			Chloroform	0.4	mL	500	mL	50.62	2500	mL	100.66	0.0161	0.0405
**Sodium acetate**	0.08	mL	3 M	19.7	mg	250	g	22.47	1000	g	56.30	0.0011	0.0018
**Isopropanol**	0.9	mL	100%	0.9	mL	1000	mL	30.30	2500	mL	61.70	0.0222	0.0273
**Ethanol**	1.5	mL	70%	1.05	mL	250	mL	47.56	2500	mL	246.58	0.1036	0.1998
**TE buffer**	0.1	mL	10 mM Tris–HCl	0.12	mg	100	g	34.08	1000	g	245.23	3.0E-05	4.1E-05
			1.0 mM EDTA	0.03	mg	500	g	93.40	1000	g	128.00	3.7E-06	5.5E-06
**Extracted samples**	12					–			–		–	–	–
**Extraction time**	210	min				–		–	–		–	–	–
**Lab staff (per hour)**						–		35.00	–		25,00	–	–
**Lab staff (€/extraction)**												**7.29**	**10.21**
**Chemicals (€/extraction)**												**0.19**	**0.38**
**Gloves, tubes, tips (€/extraction)**												**0.26**	**0.37**
**Final price per extraction (€)**												**7.74**	**10.96**

**Table 10:** Cost analysis for DNA extraction procedure I (according to Tresse et al. [6]).

**Consumables**	**Volumes**	**Units**	**Concentration**	**Volumes**	**/Weight**	**High costs**			**Low costs**			**Low cost**	**High cost**
						**Amount**	**Unit**	**Fix cost (€)**	**Amount**	**Unit**	**Fix cost (€)**	**per Prep (€)**	**per Prep (€)**
**Gloves (any size)**	1	pair	–	–	–	50	pair	8.20	50	pair	4.50	0.0900	0.1640
**Tubes**	3	–	–	2.0	mL	500	pieces	11.90	1000	pieces	21.90	0.0657	0.0714
	4	–	–	1.5	mL	500	pieces	8.20	1000	pieces	14.90	0.0596	0.0656
**Tips**	14		–	1000	µL	500	pieces	5.08	1000	pieces	7.70	0.1078	0.1421
**Tips**	4		–	200	µL	500	pieces	5.40	1000	pieces	8.19	0.0328	0.0432
**Tips**	1		–	10	µL	1000	pieces	27.14	2000	pieces	43.42	0.0217	0.0271
**TEN buffer**	0.7	mL	100 mM Tris	8.48	mg	500	g	93.40	1000	g	128.00	0.0011	0.0016
			100 mM EDTA	20.45	mg	100	g	34.08	1000	g	245.23	0.0050	0.0070
			100 mM NaCl	4.09	mg	500	g	15.84	1000	g	24.19	9.9E-05	1.3E-04
			5 mg mL-1 Lysozyme	3.5	mg	1.0	g	23.89	10	g	96.04	0.0336	0.0836
**SDS**	0.035	mL	20% w/v	0.007	mg	100	g	16.56	1000	g	56.48	4.0E-07	1.2E-06
**Proteinase K**	0.01	mL	20 mg mL-1	0.2	mg	100	mg	67.68	500	mg	259.62	0.1038	0.1354
**Silica beads**	–		ID 0.1 mm	250	mg	1000	g	24.30	25000	g	202.00	2.0E-03	0.0061
**Silica beads**	–		ID 0.5 mm	250	mg	1000	g	25.23	20000	g	227.18	0.0028	0.0063
**Silica beads**	2	beads	ID 6.0 mm	69	mg	500	g	34.20	1000	g	12.35	0.0009	0.0047
**Ammoniumacetate**	0.145	mL	10 M	111.8	mg	250	g	15.30	1000	g	45.29	0.0051	0.0068
**RNase A**	0.005	mL	1 mg mL-1	0.005	mg	250	mg	94.40	1000	mg	292.00	0.0015	0.0019
**Phenol:Chloroform:Isoamyl**	1.5	mL	25' Phenol	0.75	g	100	g	18.00	1000	g	64.40	0.0483	0.1350
**(25:24:1 v/v)**			24' Chloroform	0.72	mL	500	mL	50.62	2500	mL	100.66	0.0290	0.0729
			1' Isoamyl	0.03	mL	25	mL	13.92	1000	mL	108.00	0.0032	0.0167
**Chloroform:Isoamyl**	0.7	mL	24' Chloroform	0.672	mL	500	mL	50.62	2500	mL	100.66	0.0271	0.0680
**(24:1 v/v)**			1' Isoamyl	0.028	mL	25	mL	13.92	1000	mL	108.00	0.0030	0.0156
**Sodiumaceate (1:10 v/v)**	0.07	mL	3 M	17.2	mg	250	g	22.47	1000	g	56.30	0.0010	0.0015
**Ethanol (2:1 v/v)**	1.4	mL	98%	1.37	mL	250	mL	47.56	2500	mL	246.58	0.1353	0.2610
**Ethanol**	1.0	mL	70%	0.7	mL	250	mL	47.56	2500	mL	246.58	0.0690	0.1332
**TE buffer**	0.1	mL	10 mM Tris–HCl	0.12	mg	100	g	34.08	1000	g	245.23	3.0E-05	4.1E-05
			1.0 mM EDTA	0.03	mg	500	g	93.40	1000	g	128.00	3.7E-06	5.5E-06
**Extracted samples**	12					–			–		–	–	–
**Extraction time**	170	min				–		–	–		–	–	–
**Lab staff (per hour)**						–		35.00	–		25.00	–	–
**Lab staff (€/extraction)**												**5.90**	**8.26**
**Chemicals (€/extraction)**												**0.47**	**0.96**
**Gloves, tubes, tips (€/extraction)**												**0.38**	**0.51**
**Final price per extraction including extraction time, lab staff and all consumables (€)**												**6.75**	**9.73**

**Table 11:** Cost analysis for DNA extraction procedure J (according to Wilson [7]).

**Consumables**	**Volumes**	**Units**	**Concentration**	**Volumes**	**/Weight**	**High costs**			**Low costs**			**Low cost**	**High cost**
						**Amount**	**Unit**	**Fix cost (€)**	**Amount**	**Unit**	**Fix cost (€)**	**per Prep (€)**	**per Prep (€)**
**Gloves (any size)**	1	pair	–	–	–	50	pair	8.20	50	pair	4.50	0.0900	0.1640
**Tubes**	3	–	–	2.0	mL	500	pieces	11.90	1000	pieces	21.90	0.0657	0.0714
**Tips**	9		–	1000	µL	500	pieces	5.08	1000	pieces	7.70	0.0693	0.0914
**Tips**	4		–	200	µL	500	pieces	5.40	1000	pieces	8.19	0.0328	0.0432
**Tips**	1		–	10	µL	1000	pieces	27.14	2000	pieces	43.42	0.0217	0.0271
**TE buffer**	0.567	mL	10 mM Tris	0.69	mg	100	g	34.08	1000	g	245.23	0.0002	0.0002
			10 mM EDTA	1.66	mg	500	g	93.40	1000	g	128.00	0,0002	0.0003
**SDS**	0.03	mL	10% w/v	0.003	mg	100	g	16.56	1000	g	56.48	1.7E-07	5.0E-07
**Proteinase K**	0.003	mL	20 mg mL-1	0.06	mg	100	mg	67.68	500	mg	259.62	0.0312	0.0406
**NaCl**	0.1	mL	5 M	29.22	mg	500	g	15.84	1000	g	24.19	7.1E-04	9.3E-04
**CTAB/NaCl**	0.08	mL	0,7 M NaCl	3,3	mg	500	g	15.84	1000	g	24.19	0.0001	0.1037
			10% w/v CTAB	0.008	mg	100	g	22.64	1000	g	89.11	7.1E-07	1.8E-06
**Chloroform:Isoamyl**	1.0	mL	24' Chloroform	0.96	mL	500	mL	50.62	2500	mL	100.66	0.0387	0.0972
**(24:1 v/v)**			1' Isoamyl	0.04	mL	25	mL	13.92	1000	mL	108.00	0.0043	0.0223
**Phenol:Chloroform:Isoamyl**	0.9	mL	25' Phenol	0.45	g	100	g	18.00	1000	g	64.40	0.0290	0.0810
**(25:24:1 v/v)**			24' Chloroform	0.432	mL	500	mL	50.62	2500	mL	100.66	0.0174	0.0437
			1' Isoamyl	0.018	mL	25	mL	13.92	1000	mL	108.00	0.0019	0.0100
**Isopropanol (0.6: 1 v/v)**	0.3	mL	100%	0.3	mL	1000	mL	30.30	2500	mL	61.70	0.0074	0.0091
**Ethanol**	0.5	mL	70%	0.35	mL	250	mL	47.56	2500	mL	246.58	0.0345	0.0666
**TE buffer**	0.1	mL	10 mM Tris–HCl	0.12	mg	100	g	34.08	1000	g	245.23	3.0E-05	4.1E-05
			1.0 mM EDTA	0.03	mg	500	g	93.40	1000	g	128.00	3.7E-06	5.5E-06
**Extracted samples**	12					–		–	–		–	–	–
**Extraction time**	105	min				–		–	–		–	–	–
**Lab staff (per hour)**						–		35.00	–		25.00	–	–
**Lab staff (€/extraction)**												**3.65**	**5.10**
**Chemicals (€/extraction)**												**0.17**	**0.48**
**Gloves, tubes, tips (€/extraction)**												**0.28**	**0.40**
**Final price per extraction including extraction time, lab staff and all consumables (€)**												**4.09**	**5.98**

**Table 12:** Cost analysis for FastDNA SPIN Kit for Soil.

**Consumables**	**Volumes**	**Units**	**Concentration**	**Volumes**	**/Weight**	**High costs**			**Low costs**			**Low cost**	**High cost**
						**Amount**	**Unit**	**Fix cost (€)**	**Amount**	**Unit**	**Fix cost (€)**	**per Prep (€)**	**per Prep (€)**
**Gloves (any size)**	1	pair	–	–	–	50	pair	8.20	50	pair	4.50	0.090	0.164
**Tips**	12			1000	µl	500	pieces	5.08	1000	pieces	7.70	0.092	0.122
**Tips**	4			200	µL	500	pieces	5.40	1000	pieces	8.19	0.033	0.043
**Tips**	1			10	µl	1000	pieces	27.14	2000	pieces	43.42	0.022	0.027
**Test Kit**						50	extractions	390.00	100	extractions	820.00	8.20	7.80
**Extracted samples**	12											–	–
**Extraction time**	45	min						–			–	–	–
**lab staff (per hour)**								35.00			25.00	1.56	2.19
**Lab staff (€/extraction)**												**1.56**	**2.19**
**Chemicals (€/extraction)**												**8.20**	**7.80**
**Gloves, tubes, tips (€/extraction)**												**0.24**	**0.36**
**Final price per extraction including extraction time, lab staff and all consumables (€)**												**10.00**	**10.34**

**Table 13:** Cost analysis for DNeasy power water kit.

**Consumables**	**Volumes**	**Units**	**Concentration**	**Volumes**	**/Weight**	**Amount**	**High costs**			**Low costs**		**Low cost**	**High cost**
							**Unit**	**Fix cost (€)**	**Amount**	**Unit**	**Fix cost (€)**	**per Prep (€)**	**per Prep (€)**
**Gloves (any size)**	1	pair	–	–	–	50	pair	8.20	50	pair	4.50	0.090	0.164
**Tips**	12			1000	µl	500	pieces	5.08	1000	pieces	7.70	0.092	0.122
**Tips**	4			200	µL	500	pieces	5.40	1000	pieces	8.19	0.033	0.043
**Tips**	1			10	µl	1000	pieces	27.14	2000	pieces	43.42	0.022	0.027
**Test Kit**						50	extractions	558.61	100	extractions	1062.9	10.63	11.17
**Extracted samples**	12											–	–
**Extraction time**	40	min						–			–	–	–
**lab staff (per hour)**								35.00			25.00	1.39	1.94
**Lab staff (€/extraction)**												**1.39**	**1.94**
**Chemicals (€/extraction)**												**10.63**	**11.17**
**Gloves, tubes, tips (€/extraction)**												**0.24**	**0.36**
**Final price per extraction including extraction time, lab staff and all consumables (€)**												**12.25**	**13.47**
